# A Curious Case of Encephalopathy in a Hospitalized Patient: Round Up the Unusual Suspects

**DOI:** 10.7759/cureus.11970

**Published:** 2020-12-08

**Authors:** Michael E Boyars, Michael C Boyars

**Affiliations:** 1 Internal Medicine, University of Texas, Houston, USA; 2 Internal Medicine, University of Texas Medical Branch, Galveston, USA

**Keywords:** drug-related side effects and adverse reactions, encephalopathy, central line-associated infections (clabsi), esrd, end stage renal disease (esrd), toxic encephalopathy, altered mental state

## Abstract

This case describes a 52-year-old female who developed encephalopathy after being hospitalized with sepsis from a recently placed Permcath. A grade two decubitus ulcer was also a potential source of infection. Cefepime and Vancomycin were empirically begun, and the patient improved hemodynamically and clinically. She developed encephalopathy on day six of hospitalization. When the Cefepime was discontinued the encephalopathy promptly resolved. The causes of hospital acquired encephalopathy and potential difficulties in making this diagnosis are discussed with special emphasis on Cefepime induced encephalopathy.

## Introduction

Antibiotic associated encephalopathy is an under-recognized consequence in hospitalized patients. It can take the form of delirium, a decline in the level of consciousness, or even more profound symptoms such as tremors, clonic movements, and seizures. Accurate diagnosis is more difficult in this population since hospitalized patients frequently have acute and or chronic multi-organ system dysfunction or failure which may contribute to the symptoms and confuse the diagnosis. Patients with impaired renal function are particularly susceptible as many antibiotics are renally excreted. We present such a patient whom we recently encountered to emphasize this complication and review the differential diagnosis. 

## Case presentation

A 52-year-old woman with a significant medical history of end-stage renal disease, chronic hypertension, and type two diabetes mellitus presented to the emergency department two hours after experiencing chills and hypotension during her weekly scheduled hemodialysis. She initiated hemodialysis two weeks ago after having a permcath placed. The patient had a grade two sacral decubitus ulcer recently diagnosed and treated with clindamycin six days prior to admission. She reports one week of chills, constipation, and non-productive cough and denies any other symptoms such as shortness of breath, dysuria, chest pain, subjective fevers, nausea, vomiting, abdominal pain, or dysphagia. Blood cultures were drawn after dialysis and she was admitted the next day because of concern for sepsis from the permcath and or the decubitus ulcer, although the ulcer was clean, dry, and intact.

After blood cultures were drawn the patient was empirically begun on intravenous (IV) vancomycin and cefepime. The vancomycin was dosed after dialysis based on blood levels. The cefepime was renally dosed at cefepime 1 gram IV loading dose and 500 mg IV daily. On dialysis days it was given post-dialysis. The next day the blood cultures from the outpatient dialysis unit grew pseudomonas and streptococcal species. The vancomycin was discontinued and the permcath was removed, and tip cultured. The permcath was not replaced and so the patient did not receive hemodialysis until blood cultures were negative, five days later. The patient’s hemodynamic state improved, and the white blood cell count returned towards normal over the next few days, however on day four a new 2/6 left upper sternal border murmur was noted. There was concern for bacterial endocarditis, so a transthoracic echocardiogram was done and no vegetations or significant valvular abnormalities were detected. The patient continued to clinically improve otherwise, remaining afebrile with no leukocytosis. On the sixth hospital day, she was noted to be encephalopathic being oriented times one. No other focal neurologic signs were noted at that time. Neurologic consultation was obtained and an electroencephalogram (EEG), magnetic resonance imaging (MRI) of the brain, and lumbar puncture (LP) were performed. All showed no significant abnormalities. After reviewing these results neurology opined that this was delirium most likely due to metabolic changes from hemodialysis holiday. The patient was transferred to the intensive care unit for closer monitoring. After hemodialysis was reinstituted the patient’s mental state continued to deteriorate. The patient was oriented times zero and developed bilateral, non-synchronous clonic upper extremity jerks with an upper extremity resting tremor. Initially, the encephalopathy was thought to be due to toxic metabolic derangements due to missed hemodialysis during the five-day period without access. The newly auscultated murmur brought bacterial endocarditis into the differential diagnosis. This was later ruled out by the echocardiogram. Acute confusional state post-hospitalization was also considered. On day seven, cefepime was discontinued because the patient had completed a full course. The following day the patient’s mental state began to improve. She responded to commands and was oriented times three. Her improvement in mental state continued until by Day 11 she was at her baseline and was discharged. 

**Table 1:** Differential Diagnosis of Acute Encephalopathy in Hospitalized Patients

Differential Diagnosis of Acute Encephalopathy in Hospitalized Patients
Septic encephalopathy
Hepatic encephalopathy
Uremic encephalopathy
Hyponatremia
Hypernatremia
Hypoglycemia
Hyperosmolar hyperglycemic state
Wernicke encephalopathy
Hypoxic-ischemic encephalopathy
Post-transfusion encephalopathy
Alcohol withdrawal
Meningitis/encephalitis
Brain tumors
Traumatic brain injury
Schizophrenia
Drug and/or toxin induced

## Discussion

Encephalopathy originates from a Greek word literally translated meaning suffering inside the head [1]. Metabolic encephalopathy denotes a syndrome of temporary or permanent disruption of cerebral functions in the absence of structural brain disease. It occurs in diverse clinical settings and diseases. Clinical findings range from subtle cognitive difficulties to striking delirium or coma. Other manifestations can include confusion, fluctuation in mental state, hallucinations, seizures, tremor, asterixis myoclonus, and many more. The differential diagnosis of encephalopathy in hospitalized patients is long and varied. A partial list is given in Table one. Cefepime induced encephalopathy is a clinical diagnosis of exclusion. There are no imaging or laboratory or imaging findings that are specific for this entity. The diagnosis is entertained when a patient on Cefepime develops encephalopathy and other reasonable causes are ruled out. It is confirmed when the patient improves when cefepime is discontinued.

Our patient developed encephalopathy on day four of hospitalization and day four of cefepime. The encephalopathy was initially thought to be due to electrolyte abnormalities both intra and extracellular due to the lack of dialysis, sepsis, and/or sundowning. Over the next two days, her encephalopathy clinically worsened while her primary clinical condition improved. EEG, LP, and MRI of the brain did not show pathology. Neurology consultation felt this was delirium. Looking through the retrospectoscope we should have considered other etiologies such as drug side effects at this point. She only began to improve after the cefepime course was complete and the antibiotic stopped.

Cefepime-induced encephalopathy has been reported to occur in as many as 3% of patients who use cefepime [2]. The symptoms occur from one to ten days after beginning the drug, the average being five days. Impaired consciousness, myoclonic seizures, and non-convulsive status epilepticus are among the symptoms seen [3]. 85% of a cefepime dose is excreted unchanged by the kidneys [4]. In a patient with impaired renal function, as in our case, it can easily accumulate to toxic levels and lead to neurotoxicity. This toxicity is due to Cefepime’s ability to cross the blood-brain barrier and effect concentration-dependent gamma-aminobutyric acid antagonism [5,6]. Although guidelines for dosing cefepime in end-stage renal disease were followed in this patient, neurotoxicity still developed [7]. In a recent review, it was found that using Food and Drug Administration approved dosing guidelines, 48% of patients overdosed while 26% developed neurotoxicity despite being adequately dosed [5]. In one large review study, an incidence of cefepime induced neurotoxicity was found to be 1 in 480 courses [6]. Common sense dictates that once a patient has had an episode of cefepime induced neurotoxicity the drug should not be used again.

## Conclusions

Cefepime is a commonly used antibiotic in hospitalized patients. Drug-induced encephalopathy should be considered in all patients on cefepime who develop a change in mental state or any neurological changes, especially those with reduced renal function. Many times coexistent acute and or chronic disease may complicate the timely consideration of cefepime associated encephalopathy as in our case. If no alternative diagnosis is more likely, Cefepime should be discontinued and other appropriate therapy added.
